# Clarifying the taxonomic status of the alien species *Branchiomma bairdi* and *Branchiomma boholense* (Annelida: Sabellidae) using molecular and morphological evidence

**DOI:** 10.1371/journal.pone.0197104

**Published:** 2018-05-10

**Authors:** Michela Del Pasqua, Anja Schulze, María Ana Tovar-Hernández, Erica Keppel, Marco Lezzi, Maria Cristina Gambi, Adriana Giangrande

**Affiliations:** 1 Department of Biological and Environmental Sciences and Technologies (DiSTeBA), University of Salento, CoNISMa Unit, Lecce, Italy; 2 Department of Marine Biology, Texas A&M University at Galveston, Galveston, Texas, United States of America; 3 Universidad Autónoma de Nuevo León, Facultad de Ciencias Biológicas, Laboratorio de Biosistemática, San Nicolás de los Garza, Nuevo León, México; 4 Smithsonian Environmental Research Center, Edgewater, Maryland, United States of America; 5 ARPAT, Environmental Protection Agency of Tuscany, A.V. Costa - Laboratory Sector - U.O. Biologia, Pisa, Italy; 6 Stazione Zoologica Anton Dohrn di Napoli, Department of Integrative Marine Ecology, Villa Dohrn- Benthic Ecology Center, Punta S. Pietro, Ischia (Napoli), Italy; 7 Stazione Zoologica Anton Dohrn di Napoli, Villa Comunale, Naples, Italy; University of Iceland, ICELAND

## Abstract

This study was performed to analyse the genetic and morphological diversity of the sabellid annelid genus *Branchiomma*, with special emphasis on a taxon so far identified as *Branchiomma bairdi*. This species, originally described from Bermuda, has frequently been reported as an invader in the Mediterranean, the Atlantic and the Eastern Pacific, but recent observations have raised some taxonomic questions. Samples of this taxon were collected from five sites in the Mediterranean Sea, two sites in the original distribution area of *B*. *bairdi* in the Gulf of Mexico and four localities in the east Pacific and Atlantic Oceans where *B*. *bairdi* has been reported as invasive. The molecular results revealed a conspicuous genetic divergence (18.5% K2P) between the sampled Mediterranean populations and all the other ones that led to a re-evaluation of their morphological characters. The latter showed that the Mediterranean and extra-Mediterranean populations also differ in some discrete morphological and reproductive features. Consequently, the Mediterranean samples were re-designated as *B*. *boholense*, another non-indigenous species originally described from Philippines. *Branchiomma bairdi* and *B*. *boholense* differ in body size, development and shape of micro and macrostylodes, size of radiolar eyes and body pigmentation. Genetic diversity was high in *B*. *boholense* from the Mediterranean as well as in *B*. *bairdi* from the Gulf of Mexico, but low in *B*. *bairdi* populations outside their native range. The phylogenetic analysis revealed the presence of connections between the Mediterranean localities as well as between native and introduced *B*. *bairdi* populations that focus the attention on the Panama Canal as important passage for the introduction of the species from the Gulf of Mexico to the north-east Pacific Ocean.

## Introduction

Introductions of marine non-indigenous species (NIS) have occurred for centuries, but in the last decades the increasing maritime traffic and aquaculture activities have favoured the spread of marine species, with irreversible and devastating impact on global biodiversity [1–7]. Under this framework, biological invasions are becoming an increasingly more pressing problem. In the past decades, monitoring and biosecurity programs have been promoted both to assess the spread and abundance of NIS in a given region and to prevent and manage new invasions [8–12]. In order to achieve these purposes, a better knowledge of both the invasion process, the status and biology of the introduced species are needed. This is particularly crucial in regions with a high number of alien species such as the Mediterranean Sea that it is highly susceptible to marine bioinvasion. Recent studies [13–15] have focused the attention on the great number of alien polychaete species reported in this basin (134) whose introduction has been mainly facilitated by the enlargement of the Suez Canal and the steading increasing maritime traffic. The Mediterranean area accounted the highest number of established and invasive polychaete taxa in the world’s ocean, suggesting the need of further investigations in this area of knowledge for exhaustive regulatory programs.

Among polychaetes annelids, some species of the Sabellidae family associated with fouling communities are often considered invasive [14,16,17], especially those belonging to the genus *Branchiomma* Kölliker, 1858. Several species in this genus have been reported outside of their natural expected distribution ranges [18]. At present, the genus *Branchiomma* includes approximately 29 recognized species, most of which with an inter-tropical distribution [17,19]. Among these species, six are reported as alien worldwide [14,17,20]: *B*. *bairdi* (McIntosh, 1885), *B*. *boholense* (Grube, 1878), *B*. *curtum* (Ehlers, 1901), *B*. *lucullanum* (Delle Chiaje, 1828), *B*. *luctuosum* (Grube, 1870) and *B*. *conspersum* (Ehlers, 1887). Three of these species have been reported also in the Mediterranean area: *B*. *bairdi*, *B*. *luctuosum*, and *B*. *boholense* [14,21,22] and since their spread in the Mediterranean the biological and ecological characteristic of these species have been deeply investigated [23–30].

*Branchiomma luctuosum*, originally described from the Red Sea, was introduced in the Mediterranean in the late 1970’s [21] and it is now a common invasive species in both Eastern and Western basins [21,31–36]. By contrast, *B*. *boholense*, native to the Indo-Pacific and introduced in the Mediterranean in 1927 [22], seems to be a rare species in the Mediterranean. It was firstly reported in the basin by Knight-Jones et al. [22], who corrected material previously identified as *B*. *lucullanum*, and it was later recorded in Cyprus [34] and in South East Spain [37]. However, both these more recent findings have been later corrected and the collected specimens were considered belonging to the species *B*. *bairdi* [25,38]. After this re-identification, all the further records within the Mediterranean basin were referred to as *B*. *bairdi*. *Branchiomma bairdi*, originally described for Bermuda [39], represents the most recent *Branchiomma* species introduced in the Mediterranean [14,34] and at present, it is considered the most invasive species of this genus along the Italian coasts [25,27]. *Branchiomma bairdi* has been collected in other Mediterranean areas: Tunisian coasts [40], Balearic sea [41] and Malta [26]. Moreover, the species has been also introduced in several places around the world: Canary and Madeira islands [26,42], Gulf of California (Mazatlán) [43–47], south Mexican Pacific [48] and Australia (Cairns and Lizard Island) [18,49].

All current findings of *B*. *bairdi* in the Mediterranean suggest an introduction through the Strait of Gibraltar, although the first record for the Mediterranean concerns the oriental basin (as *B*. *boholense*) [40]. Nevertheless, pathways and times of introductions for *B*. *bairdi* in the Mediterranean as well as in the other areas around the world, are still uncertain and only a focused phylogeographic (genetic) analysis may clarify this point [26, 42].

In this regard, in the last decades, molecular tools have been proven particularly useful to improve our understanding of the causes and consequences of biological invasion [50–55]. Genetic methods offer the possibility to gain insights into many aspects of the invasion process, including a better understanding of sources and routes of invasions, the detection of cryptic diversity and the connectivity among native and introduced populations [56–62]. However, despite all the benefits provided by the molecular approach, one of the best ways to fully characterize aquatic invasions is to combine genetic and morphological approaches, as the availability of taxonomic expertise is considered the first crucial issue for dealing with the assessment and management of NIS [11,54,63–67].

This synergistic approach might be particularly useful when the species show high intraspecific phenotypic plasticity or when distinct evolutionary lineages do not show clear morphological diversity as it has been observed for the species of the genus *Branchiomma* [18].

The present study was first designed to investigate pathways of introduction of *B*. *bairdi* in the Mediterranean Sea as well as in other extra-Mediterranean invaded regions. To this end, specimens of the taxon so far identified as *B*. *bairdi* were sampled in different Mediterranean and extra-Mediterranean sites, including some localities in the original distribution range of the species and examined via amplification of the mitochondrial cytochrome c oxidase subunit I (COI). However, the molecular results suggested the presence of two different taxa between the sampled Mediterranean and extra-Mediterranean specimens. Therefore, the purpose of this study was reoriented towards a clarification of the taxonomic status of the collected *Branchiomma* species and, consequently, results from molecular analysis were integrated with a thorough morphological examination that was performed on several individuals of each studied population.

## Materials and methods

### Sample collection and preparation

**Fig 1:**
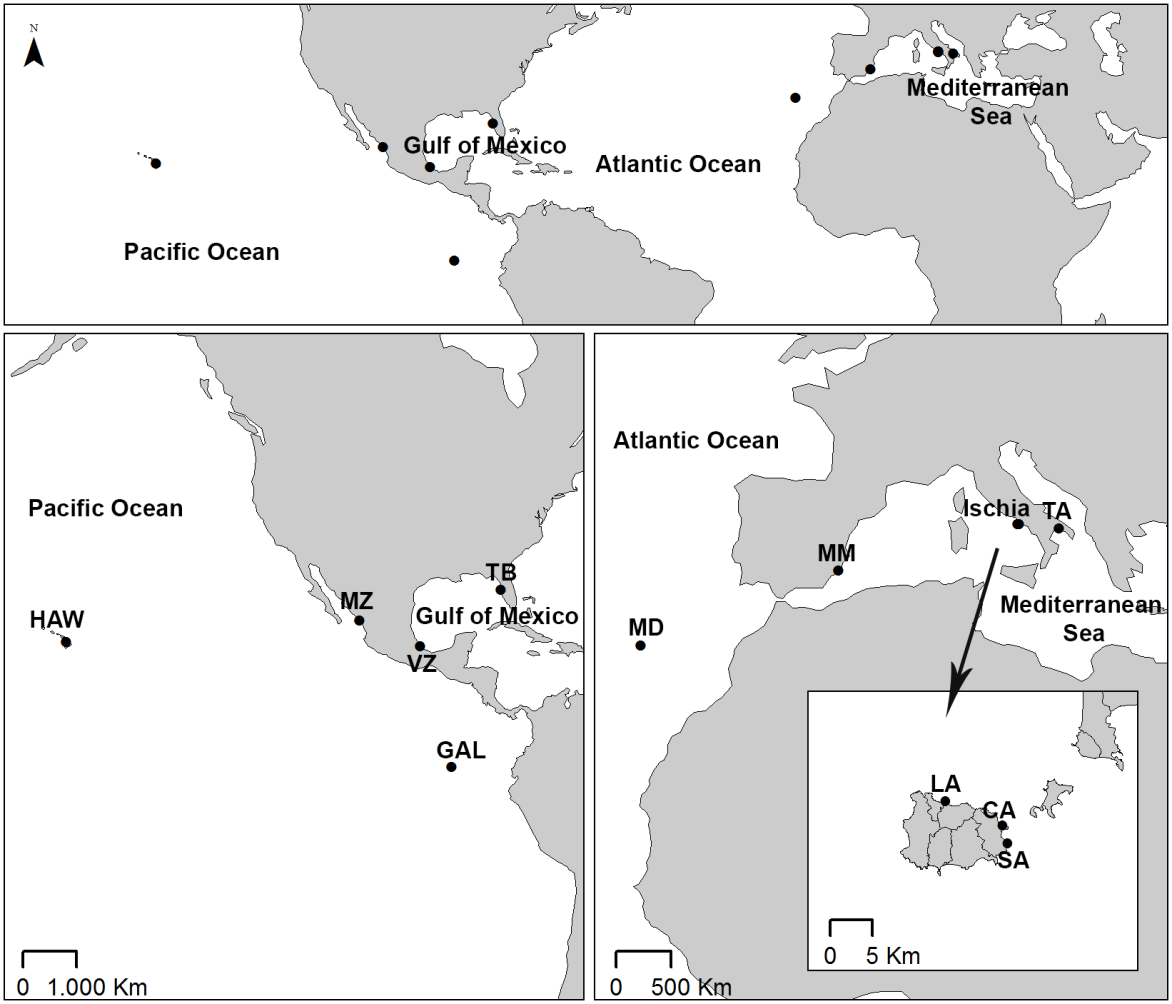
Geographical map with location of the sampling sites. For population identification see Table 1.

All the examined material was collected on hard bottom in port environments, between 1 and 5 m depth. Samples were collected between the years 2012 and 2017 and each site was only sampled once. Mediterranean specimens were collected in five sites: Mar Grande of Taranto **TA** (Ionian Sea, Italy); Ischia Island: Lacco Ameno **LA**, Sant’Anna **SA**, and Castello Aragonese **CA** (Tyrrhenian Sea, Italy); and Mar Menor **MM** (Central Mediterranen Sea, Spain). Extra-Mediterranean specimens were collected in six sites: Madeira **MD** (North East Atlantic, Portugal); Tampa Bay **TB** (Gulf of Mexico, Florida,); Veracruz **VZ** (Gulf of Mexico, Mexico,); Mazatlán **MZ** (North-East Pacific Ocean, Mexico); Galapagos **GAL** (North-East Pacific Ocean, Ecuador) and Hawaii **HW** (North-Central Pacific Ocean, USA) (Fig 1, Table 1). For Mexican material (Veracruz and Mazatlán) the sampling permission was granted by the Comisión Nacional de Acuacultura y Pesca (Authority: Lic. Aldo Gerardo Padilla Pestaño). Tampa Bay material was collected during the fouling project specifically designed to search for non-indigenous species [17,20,68]. The permission was issued by the Smithsonian Environmental Research Center (SERC) in Edgewater, Maryland. This material was part of the SERC reference collection (Authority: Dr. Gregory Ruiz). The permission for samples collection in Galapagos was allowed by the Ministry of Environment of Ecuador (MAE) and by the Galapagos National Park Directorate, while the permit for sampling specimens from Hawaii was granted by Hawaii Department of Land and Natural Resources. Sampling in Madeira island was allowed by the administrator of the Funchal Marina without any specific permission. Specimens collected from Madeira Island in our study are not considered an endangered or protected species. Taranto samples were collected in private land (aquaculture facilities Cantiere Navale Greco) and the permission was granted by the owner Dr. Francesco Grego. Samples collection from Ischia island (Lacco Ameno, Sant’Anna, Castello Aragonese) was allowed by the Ministry of the Environment and Protection of Land and Sea of Italy and by the Management Consortium of the Marine Protected Area Regno di Nettuno. The permission for sampling in Mar Menor was granted by Servicio de Pesca de la Consejería de Agricultura y Pesca (Comunidad Autónoma de la Región de Murcia).

**Table 1:** Geographic coordinates of the 11 sampling sites and estimates of genetic diversity for cytochrome c oxidase subunit I (COI) sequences (493 bp).

Sampling site	Code	Geographic Region/Country	Latitude/Longitude	N	Nh	Np	h	*π*
Taranto	TAR	SE Mediterranean Sea/ Italy	40°26'34.86"N/ 17°15'2.13"E	9	5	5	0.806±0.120	0.00297±0.0007
Lacco Ameno	LA	NW Mediterranean Sea/ Italy	40°45'10.94"N/ 13°53'26.77"E	8	5	9	0.857±0.108	0.00500±0.00124
Sant’Anna	SA	Central Mediterranean Sea/ Italy	40°43'36.66"N/ 13°57'38.15"E	14	8	9	0.824±0.098	0.00310±0.00091
Castello Aragonese	CA	Central Mediterranean Sea/ Italy	40°43'54.09"N/ 13°57'49.53"E	6	2	3	0.333±0.215	0.00203±0.00131
Mar Menor	MM	Central Mediterranean Sea/ Spain	37°40'37.39"N/ 0°47'32.93"W	11	4	4	0.782±0.075	0.00384±0.00038
Madeira	MD	East Atlantic Ocean/ Portugal	32°38'34.47"N/ 16°54'43.50"W	14	1	0	0.000±0.000	0.000±0.000
Mazatlán	MZ	NE Pacific Ocean/ Mexico	23° 16' 12.14''N/ 106° 27' 17.25''W	15	1	0	0.000±0.000	0.000±0.000
Galapagos	GAL	NE Pacific Ocean/Ecuador	0°44'52.04"S/ 90°18'44.98"W	20	2	13	0.100±0.088	0.00264±0.00232
Hawaii	HAW	NC Pacific Ocean/ USA	21°22'13.66"N/ 157°56'14.96"W	2	1	0	0.000±0.000	0.000±0.000
Veracruz	VZ	Gulf of Mexico/ Mexico	19° 11' 17.52''N/ 96° 7' 19.26''W	11	4	14	0.709±0.099	0.01328±0.00159
Tampa Bay	TB	Gulf of Mexico/ Florida	27°43'25.85"N/ 82°40'57.62"W	13	6	13	0.769 ± 0.089	0.00557 ± 0.00251
Total				123	11	44	0.618 ± 0.035	0.07322 ± 0.00293

Each sampling site refers to a different population. Sample size (N), number of haplotypes (Nh), number of polymorphic sites (Np), haplotype (h) and nucleotide (*π*) diversity for each studied population and in total.

Collected worms used for the molecular analysis were preserved in absolute ethanol and stored in the laboratory at -20°C until processing. Some specimens were fixed in 4% buffered formaldehyde seawater solution and then preserved in 70% ethanol for morphological analysis.

In addition, seven specimens of *B*. *boholense* [22] from Indonesia (Komodo Island, Sumba and Banda Sea) were morphologically examined and measured for comparative purposes. This material was collected on sandy calcareous bottom from 10 to 90 m depth in 1984 during the Indonesian-Dutch Snellius II Expedition (Zoological Museum of Amsterdam).

### Molecular analysis

DNA extraction was carried out following the procedure used by [18] using tissue removed from the posterior end of the worms. Genomic DNA was extracted using standard protocols for the DNeasy Blood and Tissues Kit (QIAGEN Pty Ltd, Dusseldorf, Germany) and stored at -20°C until further processing. The primer cocktail designed by [69] was used for the amplification of the COI gene for all the individuals of the Mediterranean populations (MM, TA, LA, SA, CA) and for some specimens collected from the Mazatlán populations (MZ). The amplifications were carried out in a 25 μl reaction containing 1 μl of the genomic DNA, 0.5 μl of HotStartIt Taq DNA polymerase, 1.5 μl of MgCl_2_ (25 mM), 1 μl of dNTPs (50 mM each), 5.5 μl of ddH_2_O, 12.5 μl of 10% trehalose, 2.5 μl of 10X buffer HotStartIt and 0.25 μl of each primer (10 mM). The PCR thermal cycling profile was 94°C for 1 min, 5 cycles of 94°C for 30s, 45°C for 40s, 72°C for 1min followed by 35 amplifications cycles of 94°C for 30s, 50°C for 40s, 72°C for 1min with a final extension step at 72°C for 10 min.

As the PCR using the COI primer cocktail [69] was unsuccessful for the amplification of the populations of Madeira, Veracruz, Galapagos, Tampa Bay and Hawaii, a new set of primers PolyBr_COIF/PolyBr_COIR was designed: the forward primer 5’ TCWATWAGWGTWATTATTCGKGCTG 3’ and the reverse 5’ CMGCAGGATYAAARAACCTAGTA 3’. PCR mixture (20 μl in total volume) contained 0.5 of the genomic DNA, 0.08 μl f Platinum Taq DNA polymerase, 0.6 μl of MgCl_2_ (50 mM), 0.4 μl dNTPs (50 mM), 15.42 μl of ddH2O, 2 μl of 10X buffer and 0.5 μl of each primer (10 mM). The PCR thermocycler protocol was 94°C for 1 min, 5 cycles at 94°C for 30 s, 58°C for 30 s, and 72°C for 40 s, 30 cycles at 94°C for 30 s, 55°C for 30 s and 72°C for 40 s, final extension at 72°C for 7 min.

PCR products were verified by means of electrophoresis on 1% agarose gel with ethidium bromide. The products of successful amplifications were purified using the ExoSAP-IT PCR purification system (USB Corporation, Cleveland, Ohio, USA) and then bidirectionally sequenced using the forward and reverse primer M13 for the Mediterranean and Mazatlán populations. For the sequencing of the samples collected from all the other populations, the new set of primer was utilized. Cycle sequencing was performed using BigDye Terminator version 3.1 in a 10 μl reaction containing 5 μl of 10% trehalose, 2 μl of 5X BigDye buffer, 1 μl of BigDye, 1 μl of each primer (or primer cocktail) and 1 μl of the PCR products. After clean-up of the sequence reactions using Zymo Sequencing Clean-Up Kit, the sequences were analysed with an ABI PRISM^®^ 3130 Genetic Analyzer [http://dx.doi.org/10.17504/protocols.io.nx6dfre]. A total of 123 sequences were generated using either of the two primer combinations (S4 Table).

Forward and reverse sequences were combined and manually edited with Sequencher v. 4.8 (Gene Codes Corporation, Ann Arbor, MI, USA). The sequences were aligned using the algorithm CLUSTAL W implemented in MEGA 5 [70] and trimmed to a uniform length of 493 bp.

The levels of genetic polymorphism for each studied population were estimated by the number of haplotypes (Nh), haplotype diversity (h) and nucleotide diversity (π) using DNAsp 5.10.01 [71].

Pair-wise F_*ST*_ among all populations was calculated using ARLEQUIN v. 3.5.2.2 [72]. The significance of the variance components and pairwise ΦST values were assessed by a permutation test with 10,000 replicates. The average sequence divergence between inferred clades was estimated using the Kimura two-parameter (K2P) model as implemented in MEGA 5 [70]. The genetic relationships among haplotypes were analyzed by a median-joining (MJ) network using the software PopART [73]. A Bayesian consensus tree was constructed with the software MrBayes v. 3.2.6 [74] using the General Time Reversible (GTR) model of evolution with gamma distributed rate variation among sites, which was selected as the optimal substitution model of sequence evolution by using the Bayesian information criterion (BIC) implemented in jModelTest 2.1.20 [75]. The Bayesian tree was estimated after 2 million generations sampled every 1000 generations. Sequences from *Bispira manicata* (Grube, 1878) [76], *Bispira serrata* Capa, 2007 [76], *Branchiomma sp*. [77] and *B*. *bairdi* [78] were used as outgroup (GenBank^®^ KX894904.1; KX894907.1; EU835657.1; HQ015126.1; KP254646.1). *Branchiomma* sp. sequences belong to specimens collected in Australia (Darwin Harbor) and Hawaii (Kaneohe Bay, Oahu) [77], while *B*. *bairdi* was collected in Florida [78].

### Morphological analysis

For this study, detailed observations of the external morphology of specimens collected from different localities were undertaken using light microscopy. For each locality, 15 adult individuals were examined and for all the specimens, the total body length (including radiolar crown, thorax and abdominal regions), the trunk length (only thorax and abdominal areas) and the radiolar crown lengths were measured. Moreover, some morphological features such as length of radiolar tips, shape of stylodes, dorsal lips and number of rows of teeth above the main fang of thoracic and abdominal uncini were analysed. The number of thoracic and abdominal chaetigers and the ratio of crown-trunk lengths were also measured.

All the analysed morphological features were photographed using a digital camera attached to the stereo- and compound microscopes (Nikon, Coolpix 990 and Carl Zeiss AxioCam Erc 5s, Zen 2012, Carl Zeiss Microscopy GmbH, Jena, Germany). Thoracic uncini were drawn with the aid of a camera lucida. Due to the variation in size and morphology of uncini along the body segments and within the tori, their morphology was analysed considering the dorsal most uncini of second thoracic chaetiger.

A one-way analysis of variance (ANOVA) was performed by using STATSOFT STATISTICA v. 6.0 to test for significant differences in the mean value of the crown/trunk length ratio between Mediterranean and extra-Mediterranean samples. Pearson correlation analysis was carried out on the number of chaetigers and the values of body length in order to evaluate the relationship between the two measurements taken.

## Results

### Genetic analysis

The sequencing of the 493 base pair fragment of the COI gene in 123 individuals identified 11 haplotypes. These haplotypes derived from 44 polymorphic sites of which 43 were parsimony informative. Most polymorphisms were the results of synonymous changes (43 of the 44 sites) while five mutations produced amino acid replacements (non-synonymous changes). Seven of the 11 identified haplotypes were location private, six of which were represented by a single individual. Four haplotypes were shared by individuals collected from distinct populations and sampling sites, two of which resulted the most common haplotypes: haplotype 1 was present just in the Mediterranean Sea and was shared by 35 individuals belonging to all the sampled Mediterranean populations, while haplotype 9 was shared by 67 individuals collected from different populations in both the Pacific Ocean, the Gulf of Mexico and the Atlantic Ocean (S1 Table).

Haplotype (h) and nucleotide (π) diversity were quite different within populations (Table 1). Haplotype diversity was high in most of the Mediterranean populations (mean h ± standard deviation = 0.806 ± 0.218), while samples from Madeira, Mazatlán, Galapagos and Hawaii populations showed low values of haplotype diversity ranging from 0.000± 0000 to 0.100±0.088. Similarly, the mean nucleotide diversity of the Mediterranean samples was high (0.0031 ± 0.001), while Mazatlán, Madeira and Hawaii populations showed minimum values of nucleotide diversity, ranging from 0.000 ± 0.000 to 0.00155 ± 0.00067, highlighting the higher genetic diversity observed in the Mediterranean samples. By contrast, the maximum values of nucleotide diversity were detected in Veracruz populations with h = 0.01328 ± 0.00159 (Table 1).

Values of pairwise Φ_ST_ and average K2P genetic distance analysis evidenced a great molecular divergence between the Mediterranean populations and those sampled both in the Gulf of Mexico, Atlantic Ocean and Pacific Ocean (S2 and S3 Tables). Indeed, all the comparison between the sampled Mediterranean populations and the Pacific, Mexican and Atlantic ones showed high and significant pairwise Φ_ST_ values, ranging from 0.93957 to 0.99684. By contrast, low and non-significant genetic divergence was observed in all the comparisons among the Mediterranean populations, as well as between the Pacific populations and the Atlantic ones, including populations from the Gulf of Mexico. Moreover, no molecular differentiation was observed in all the comparisons between Madeira, Mazatlán, Hawaii and Tampa Bay populations, while slightly higher values were obtained for the comparisons between Veracruz population and Madeira, Mazatlán, Hawaii, Galapagos and Tampa Bay (S2 Table). Comparably, values of the average Kimura two-parameter distance calculated between the different populations, evidenced a strong genetic variation (18.5% ± 0.000614) between the Mediterranean populations compared to all the other ones, while comparisons between the Mediterranean populations as well those independently calculated within the Pacific, Mexican and Atlantic group of populations showed no significant average genetic distance (S3 Table).

**Fig 2:**
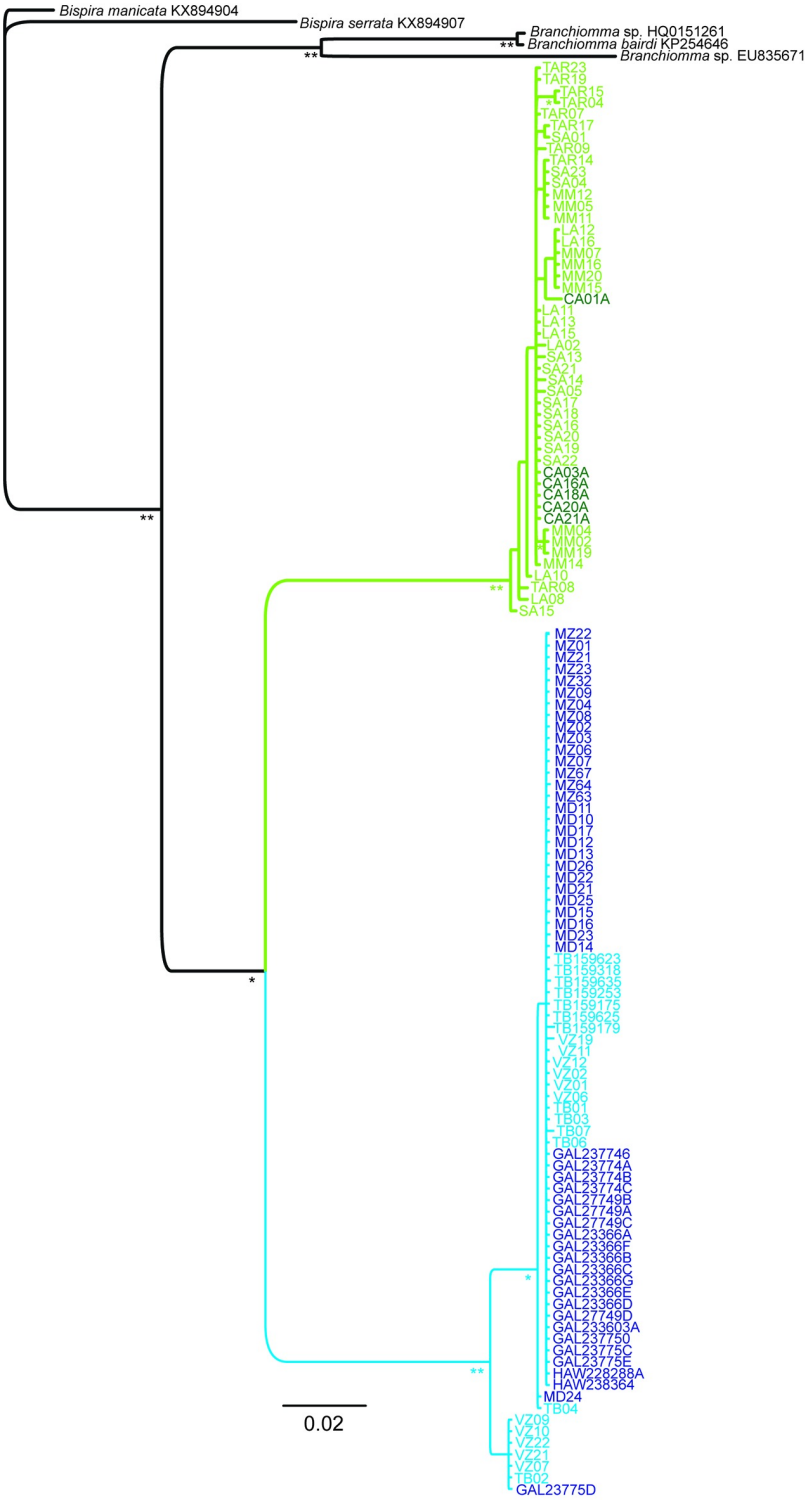
Bayesian phylogenetic tree of the 124 COI *Branchiomma* sequences obtained (marked in blue and green). OTUs names consist of the abbreviated location name according to Table 1 and a unique identifier. For Mediterranean populations, dark green indicates samples collected from carbon dioxide vents, light green samples from non-acidified sites. For extra-Mediterranean populations, dark blue indicates samples from sites where the species was introduced, light blue indicates samples from the native range. The tree was rooted using sequences of *Bispira manicata*, *Bispira serrata*, *Branchiomma sp*. and *Branchiomma bairdi* as outgroups (taxon names followed by GenBank^®^ accession numbers). Only nodes supported by posterior probabilities ≥ 0.95 are reported. **, posterior probability = 1; *, posterior probability ≥ 0.95 and < 1.

The phylogenetic tree constructed using the Bayesian approach evidenced two distinct monophyletic groups of samples (posterior probability, pp. 1.00) (Fig 2). Group 1 was well supported (pp. 1.00) and enclosed samples belonging to populations collected in the Gulf of Mexico, Pacific Ocean and Atlantic Ocean. On the other hand, Group 2 comprised exclusively COI sequences obtained from individuals collected in the Mediterranean basin (pp. 1.00). The COI sequence identified as *Branchiomma bairdi* [78] and used as outgroup did not cluster inside the studied clades of samples, probably because it refers to another *Branchiomma* species.

**Fig 3:**
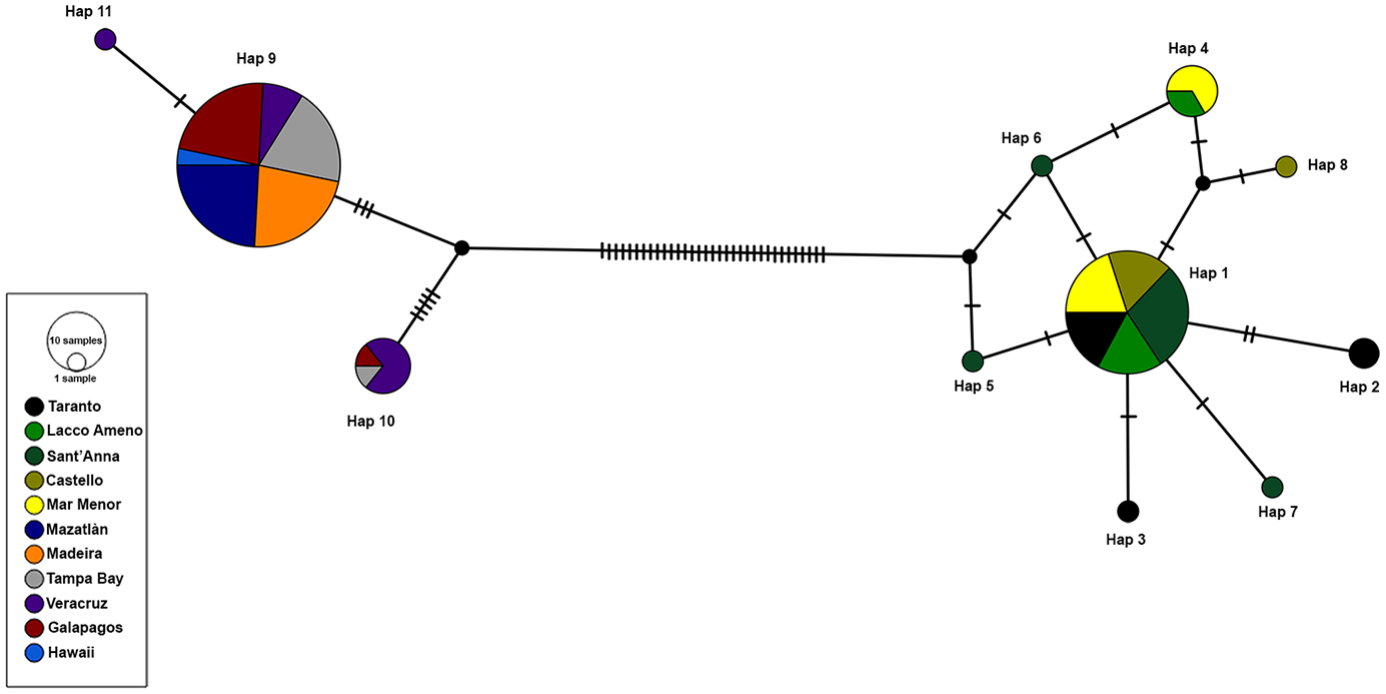
Median-joining network of haplotypes detected from the 11 studied localities. The area of each circle is proportional to the number of individuals exhibiting that haplotype (Hap). Each line joining haplotypes represents one mutational step, transversal bars show additional steps.

The MJ network of haplotype showed results highly concordant with the phylogenetic analysis. The analysis distinguished two groups of haplotypes, separated by 33 mutations (Fig 3). Haplogroup 1 included three haplotypes (Hap_9, Hap_10, Hap_11), of which one (Hap_9) was shared by the 89% of the individuals sampled from different populations in both the Gulf of Mexico, Pacific and Atlantic areas. Haplotype 10 was present in seven individuals sampled from Veracruz, Galapagos and Tampa Bay populations, while haplotype 11 was represented by a single individual and was location private. Haplogroup 2 contained eight haplotypes exclusively present in the Mediterranean basin. Haplotype 1 resulted the most frequent haplotype inside the basin, as it was shared by 35 individuals belonging to all the sampled Mediterranean populations. Haplotype 6 differentiated from haplotype 1 by one mutation and was present in four individuals, two sampled in the locality of Lacco Ameno and two belonging to Mar Menor population. Haplotype 2 was present just in the Taranto population while all the other haplotypes were location private and represented by a single individual (Fig 3, S1 Table).

### Morphological analysis

The two groups of populations that molecular analysis showed to be genetically divergent, also showed slight morphological differences. As shown in Table 2, one of the main differences concerns body size. Individuals from Mediterranean populations showed higher values of total body length (mean total length ± standard deviation = 3.3 ± 0.7 cm) than extra-Mediterranean specimens (1.85 ± 0.6 cm). Likewise, Mediterranean specimens had higher values of trunk length (mean trunk length ± standard deviation = 2 ± 0.6 cm) and crown length (mean crown length ± standard deviation = 1.3 ± 0.2 cm) than extra-Mediterranean ones (1.25 ± 0.5 cm, 0.5 ± 0.1 cm). Specimens from Indonesia showed intermediate values of total body length (2.44 ± 0.7 cm), trunk length (1.66 ± 0.6 cm) and crown length (0.77 ± 0.12 cm) between Mediterranean and extra Mediterranean populations.

**Table 2:** Mean values of total body length, trunk length, crown length and crown/trunk length ratio for each Mediterranean and extra-Mediterranean studied localities.

	Mean total body size (cm)	Mean trunk length (cm)	Mean crown length (cm)	Mean CL/TL (cm)
Taranto	3.80 ± 0.83	2.60 ± 0.68	1.30 ± 0.25	0.52 ± 0.20
Sant’Anna	3.00 ± 0.70	1.70 ± 0.56	1.30 ± 0.21	0.66 ± 0.14
Lacco Ameno	3.20 ± 0.61	2.00 ± 0.39	1.20 ± 0.22	0.61 ± 0.08
Castello Aragonese	3.10 ± 0.72	2.00 ± 0.57	1.20 ± 0.25	0.58 ± 0.12
Mar Menor	3.70 ± 0.69	2.30 ± 0.56	1.40 ± 0.17	0.64 ± 0.14
**Total**	**3.30 ± 0.77**	**2.00 ± 0.63**	**1.30 ± 0.21**	**0.60 ± 0.14**
Mazatlán	1.70 ± 0.51	1.00 ± 0.41	0.60 ± 0.13	0.44 ± 0.11
Tampa Bay	1.80 ± 0.61	1.30 ± 0.56	0.50 ± 0.10	0.41 ± 0.15
Veracruz	2.10 ± 0.48	1.30 ± 0.38	0.60 ± 0.18	0.46 ± 0.17
Galapagos	2.00 ± 0.51	1.40 ± 0.41	0.50 ± 0.13	0.40 ± 0.10
Hawaii	1.30 ± 0.29	0.90 ± 0.24	0.40 ± 0.11	0.54 ± 0.18
Madeira	2.40 ± 0.60	1.70 ± 0.52	0.70 ± 0.17	0.36 ± 0.14
**Total**	**1.85 ± 0.60**	**1.25 ± 0.50**	**0.50 ± 0.16**	**0.42 ± 0.15**

**Fig 4:**
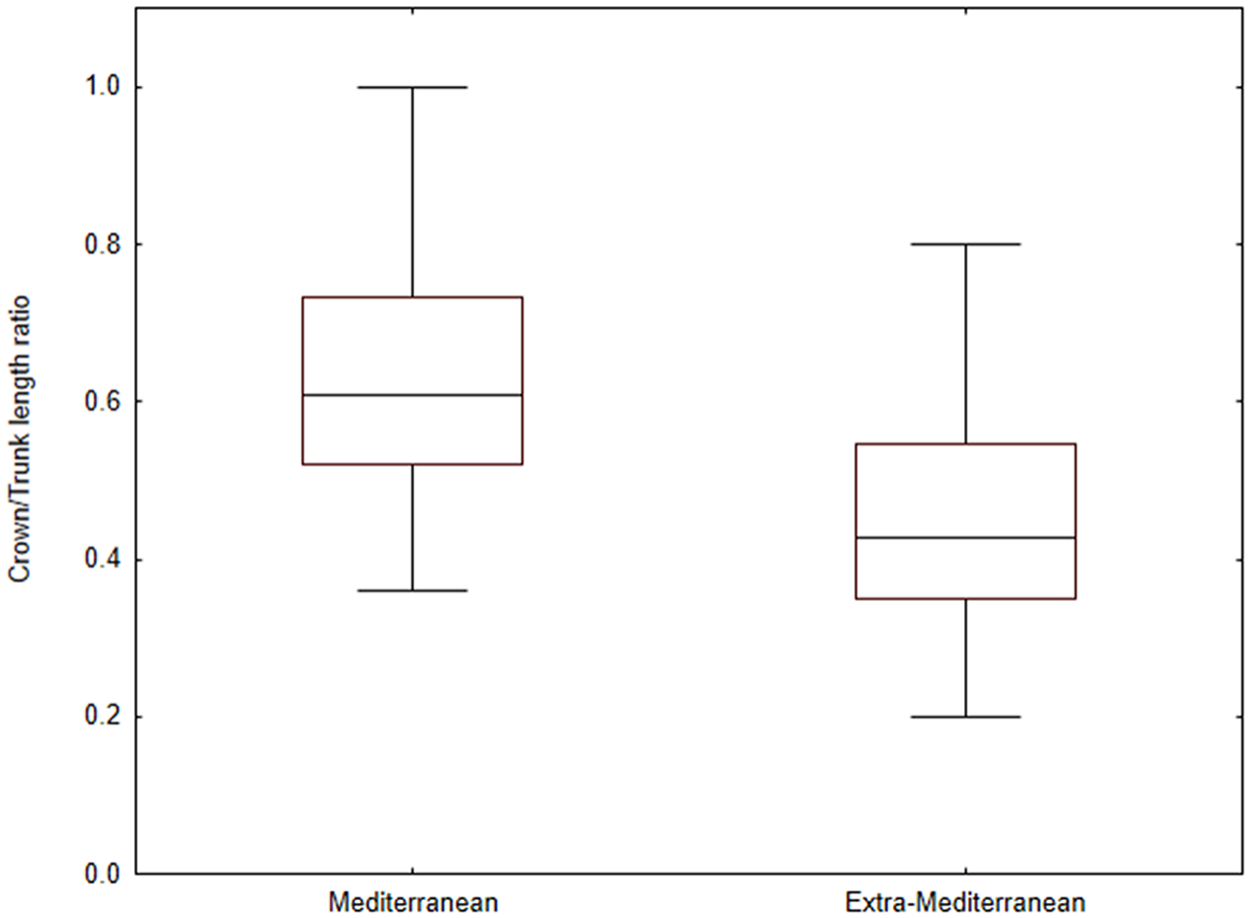
Box-plot graph of the ANOVA results. The graph shows the values of crown/trunk length ratio reported for Mediterranean and extra-Mediterranean specimens. Differences in these values were tested using crown/trunk length ratio as dependent variable and the geographical origin of the samples (Mediterranean, extra-Mediterranean) as factor.

**Fig 5:**
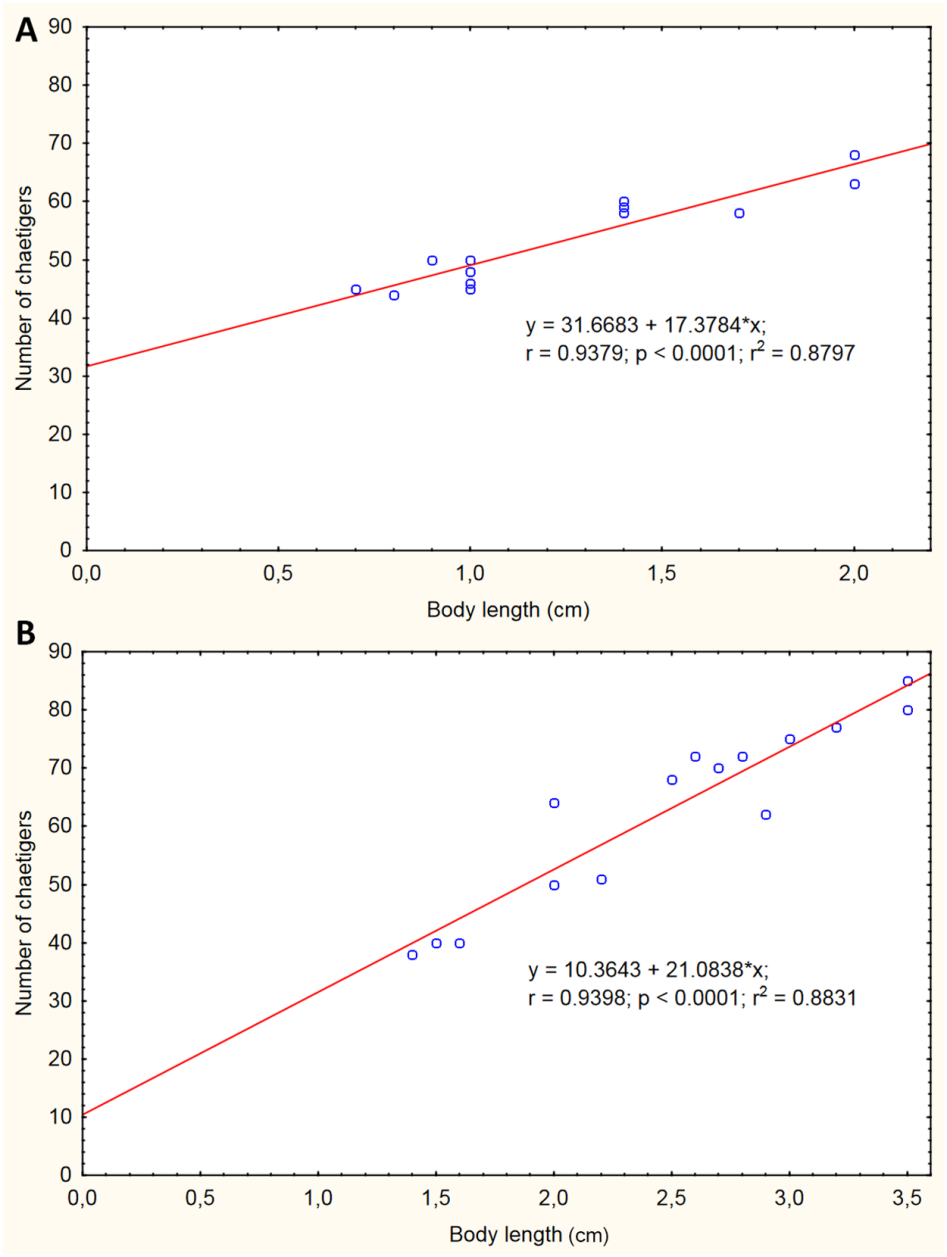
Relationship among biometrical measurement in the studied *Branchiomma* samples. The graphs show the correlation between individuals’ total body size and number of chaetigers for Mediterranean and extra-Mediterranean populations. (A) Trunk length-number of chaetigers relationship for Mediterranean samples (Taranto population). (B) Trunk length-number of chaetigers relationship for extra-Mediterranean samples (Mazatlán).

Moreover, significant differences (p<0.0001) were detected in the values of crown/trunk length ratio between Mediterranean and non-Mediterranean samples (Table 3). The values of crown/trunk length ratio resulted higher in Mediterranean populations than in extra-Mediterranean samples (Fig 4). Results from Pearson correlation analysis evidenced a linear relationship between body size of the worms and the number of chaetigers both in Mediterranean (Fig 5A) and extra-Mediterranean samples (Fig 5B).

**Table 3:** Results of the ANOVA analysis testing for differences in average crown/trunk length ratio measured in Mediterranean and extra-Mediterranean samples.

Source of variation	Df	MS	F	p
Mediterranean vs extra-Mediterranean	1	49.43971	289.305	**< 0.0001**

Df: degrees of freedom, MS: mean square, F: F-statistics, p: P-values. Significant p values are in bold.

**Fig 6:**
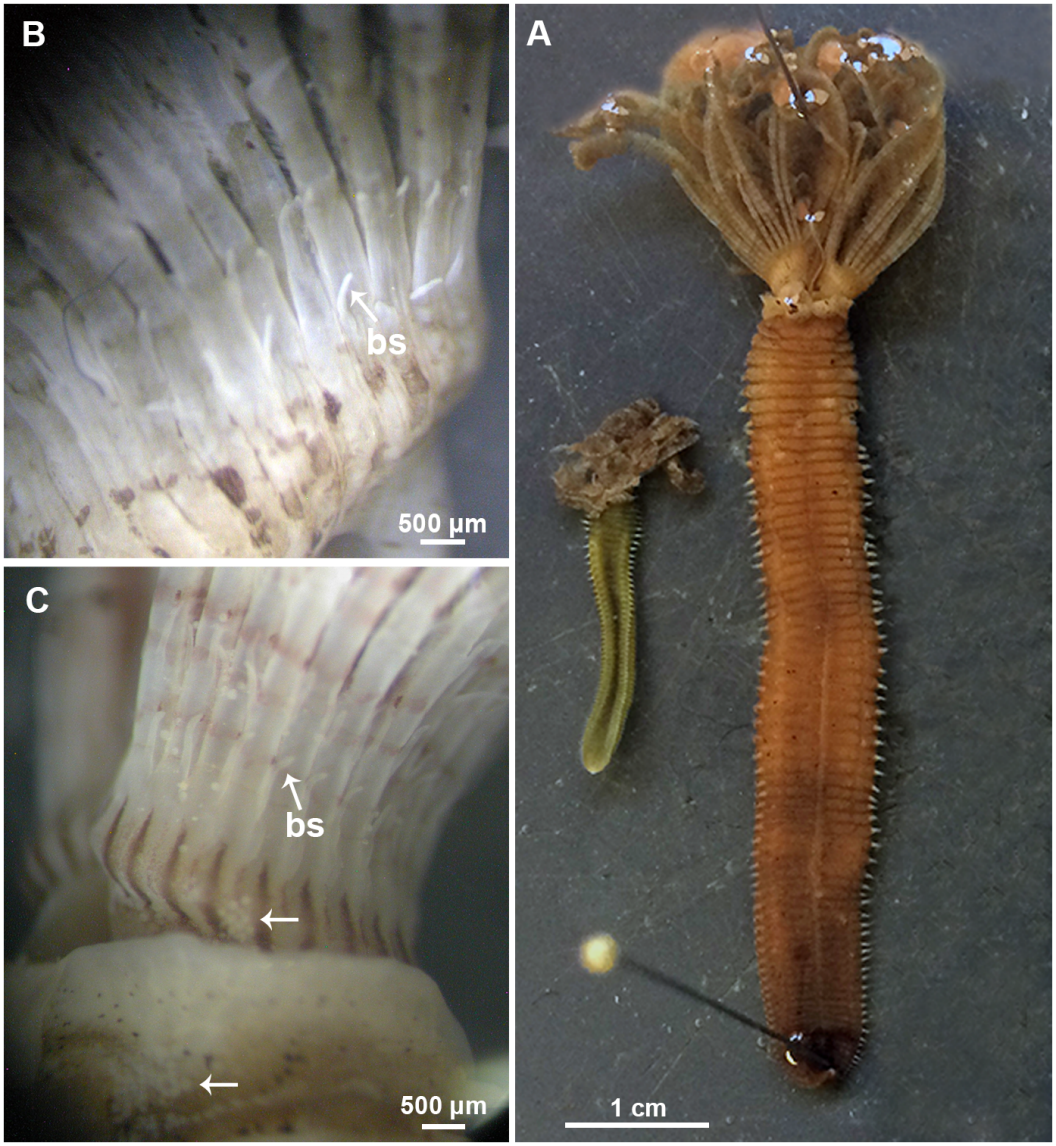
Morphological comparison of Mediterranean (Taranto) and extra-Mediterranean (Mazatlán) *Branchiomma* samples. (A) Complete specimens in ventral view; r: Mediterranean specimen, l: extra-Mediterranean specimen. (B) Mediterranean specimen. Basal part of the crown with the detail of the first pair of stylodes. (C) Extra-Mediterranean specimen. Basal part of the crown with the detail of the first pair of stylodes. The arrow indicates eggs masses.

**Fig 7:**
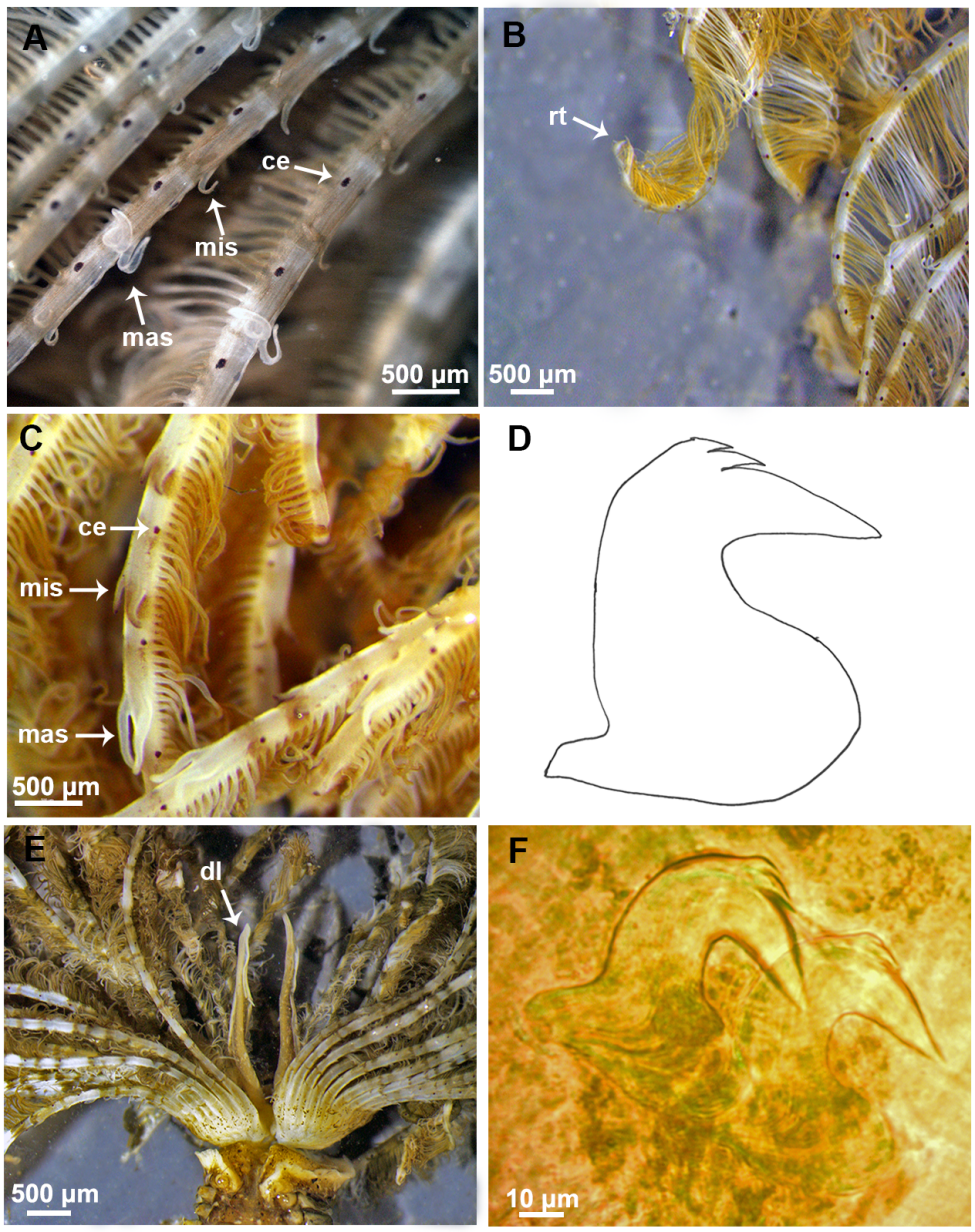
Morphological features of a Mediterranean specimen. (A) Detail of the radioles with stylodes, macrostylodes and radiolar eyes. (B) Detail of the radiolar tips. (C) Macrostylodes and microstylodes. (D) Drawn of thoracic avicular uncinus. (E) Dorsal lips. (F) Thoracic avicular uncini. mis: microstylodes, mas: macrostylodes, ce: compound eyes, rt: radiolar tips, dl: dorsal lips.

**Fig 8:**
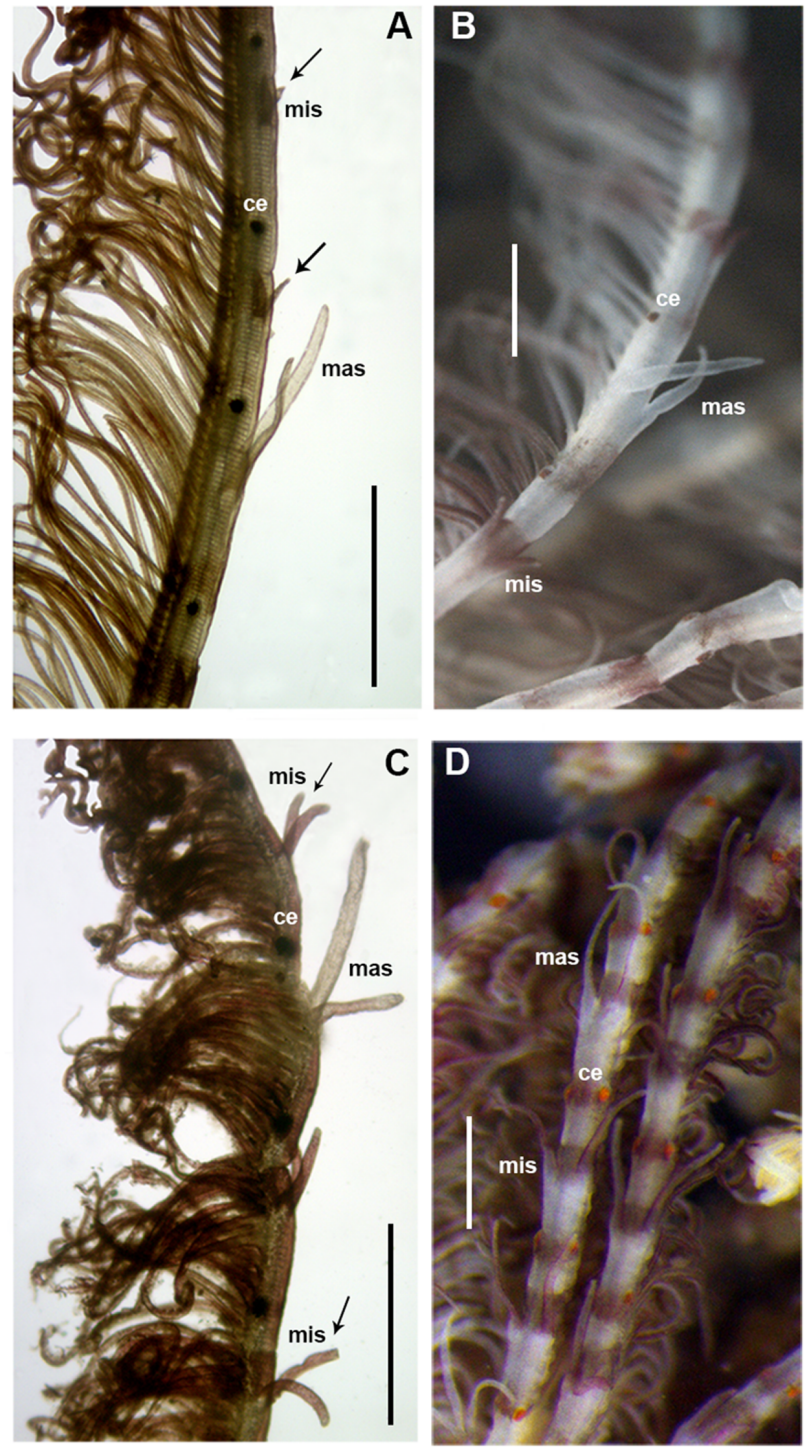
Morphological comparisons of branchial radioles for Mediterranean (Taranto) and extra-Mediterranean (Mazatlán) specimens. Details of radioles with microstylodes, macrostylodes and radiolar eyes from Mediterranean (A, B) and extra-Mediterranean (C, D) samples. mis: microsylodes, mas: macrostylodes, ce: compound eyes. Arrows highlight the different length of microstylodes between Mediterranean and extra-Mediterranean samples. Scale bars: A-D, 500 μm.

The morphological features of Mediterranean and extra-Mediterranean specimens are listed in Table 4. Individuals from Mediterranean material showed a brownish colouration with small dark spots both in preserved and live specimens (Fig 6A). Their radiolar crown showed to bear from 20 to 25 radioles with short radiolar tips (as long as equivalent space of 6–8 pinnules), in some specimens abruptly tapering with pinnules extending up to the radiolar tips (Fig 7B). Dorsal lips were as long as 1/3 of the crown (Fig 7E). Microstylodes appeared digitiform in all the examined individuals, small, as long as rachis width or slightly longer (Figs 7A, 7C and 8A, 8B) and not covering the radiolar eyes that appeared small (covering less than 1/2 of side of radiolar rachis at mid-radiolar length) along the radiole and brownish coloured (Figs 7A, 7C and 8A, 8B). Flattened, tongue-like macrostylodes, 2–3 times longer than microstylodes, were present in number of 1–4 pairs per radiole (Figs 7A, 7C and 8A, 8B). However, high variability in the morphology of macrostylodes was observed according to the position of the radiole within the radiolar crown, indeed, some macrostylodes appeared strap-like (cylindrical). Variation was also viewed in tips of macrostylodes, sometimes being distally rounded others being acuminate (Fig 8A and 8B). The basal microstylodes appeared small (as long as rachis width or slightly longer) and unpaired (Fig 6B). Thoracic and abdominal uncini with 1–2 rows of teeth over the main fang (Fig 7D and 7F).

**Table 4:** Morphological features of Mediterranean (*B*. *boholense*) and extra-Mediterranean (*B*. *bairdi*) specimens.

Morphological features	Mediterranean taxon (presumably *B*. *boholense*)	Extra-Mediterranean taxon (*B*. *bairdi*)
numbers of radioles	20–25 pairs	15–18 pairs
basal stylodes	small (as long as rachis width or slightly longer), unpaired	long (notoriously longer than rachis width), unpaired
shape of macrostylodes	flattened, tongue-like mainly but strap-like may be present	cylindrical, strap-like
shape and size of microstylodes	short (as long as rachis width or slightly longer), digitiform	long (notoriously longer than rachis width), digitiform
compound eyes	small (covering less than 1/2 of side of radiolar rachis at mid-radiolar length) /brownish	big (covering 3/4 of side of radiolar rachis at mid-radiolar length)/orange
radiolar tips	short (as long as equivalent space of 6–8 pinnules), tapering	long (occupying the space of 10–15 pinnules)
dorsal lips	1/3 of the crown	1/3 of the crown
body pigmentation	dark brownish with small dark spots (preserved and alive)	yellow-green (alive)/brownish (preserved) with small brownish spots
body size	Medium (2–2.5 cm), large-sized (superior to 3 cm)	Small (1–1.9 cm), medium-sized (2–2.5 cm)
number of teeth over the main fang of thoracic uncini	1–2	2–3

**Fig 9:**
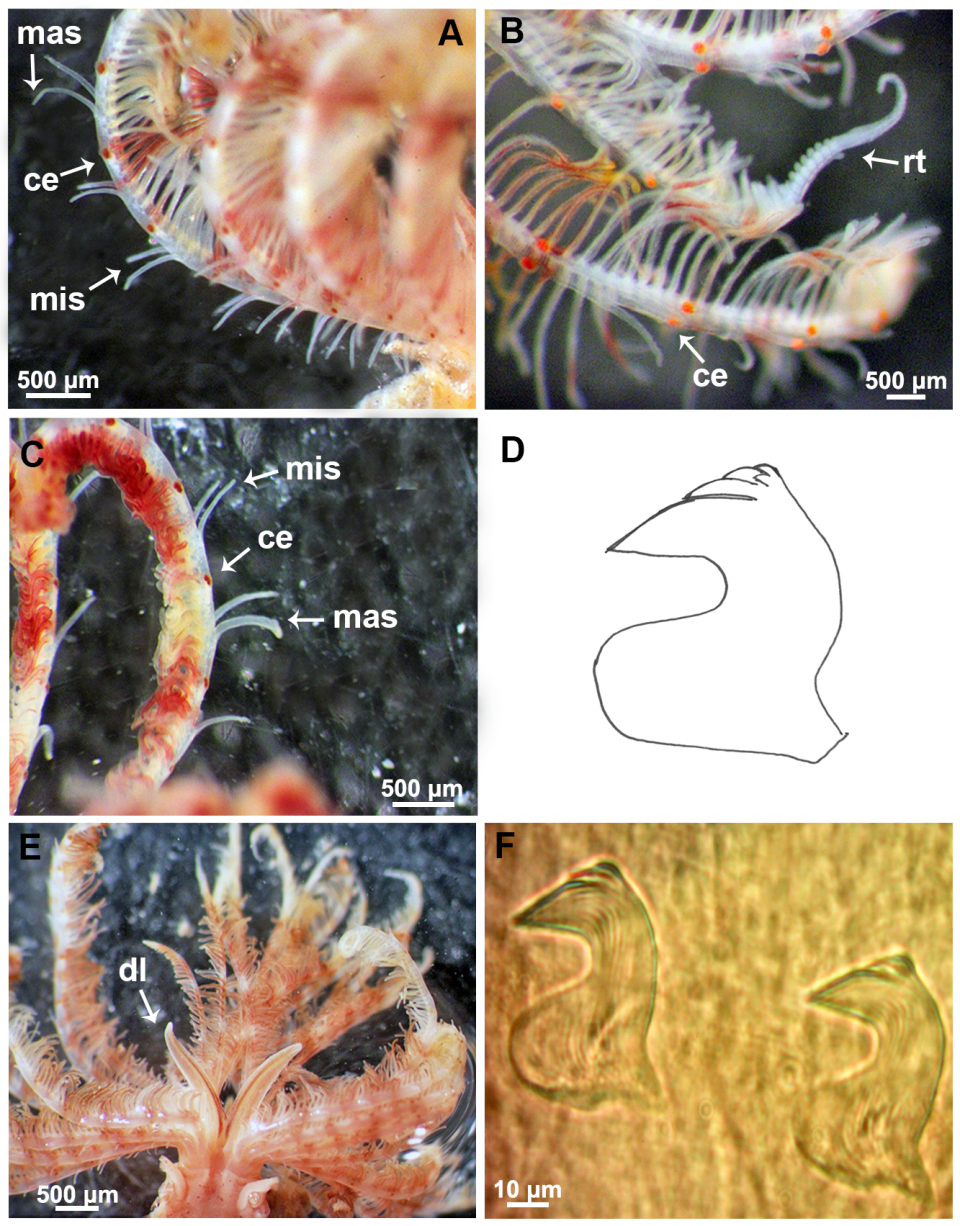
Morphological features of an extra-Mediterranean specimen. (A) Detail of the radioles with stylodes, macrostylodes and radiolar eyes. (B) Detail of the radiolar tips. (C) Macrostylodes, microstylodes and radiolar eyes. (D) Drawing of thoracic avicular uncinus. (E) Dorsal lips. (F) Thoracic avicular uncini. mis: microstylodes, mas: macrostylodes, ce: compound eyes, rt: radiolar tips, dl: dorsal lips.

Specimens from all the extra-Mediterranean material showed a green (in alive specimens) and brownish colouration (in fixed specimens) with interramal small brownish spots along the body and an orange-red pigmented crown in some specimens (Fig 6A). Their radiolar crown showed from 15 to 18 pairs of radioles terminating with long radiolar tips, as long as about 10–15 pairs of pinnules (Fig 9B). Several specimens showed the presence of the eggs within the crown, suggesting brooding (Fig 6C). Dorsal lips were as long as 1/3 of the crown (Fig 9E). The microstylodes appeared long (notoriously longer than rachis width), thin and digitiform in all the examined individuals, but not covering the radiolar eyes that appeared highly developed (covering 3/4 of side of radiolar rachis at mid-radiolar length) and orange coloured (Figs 8D and 9A, 9C). Macrostylodes were strap-like, 2–3 times longer than microstylodes and 1–4 pairs per radiole (Figs 8C, 8D and 9C). The basal microstylodes appeared unpaired (Fig 6C). Thoracic and abdominal uncini with 2–3 rows of teeth over the main fang (Fig 9D and 9F).

**Fig 10:**
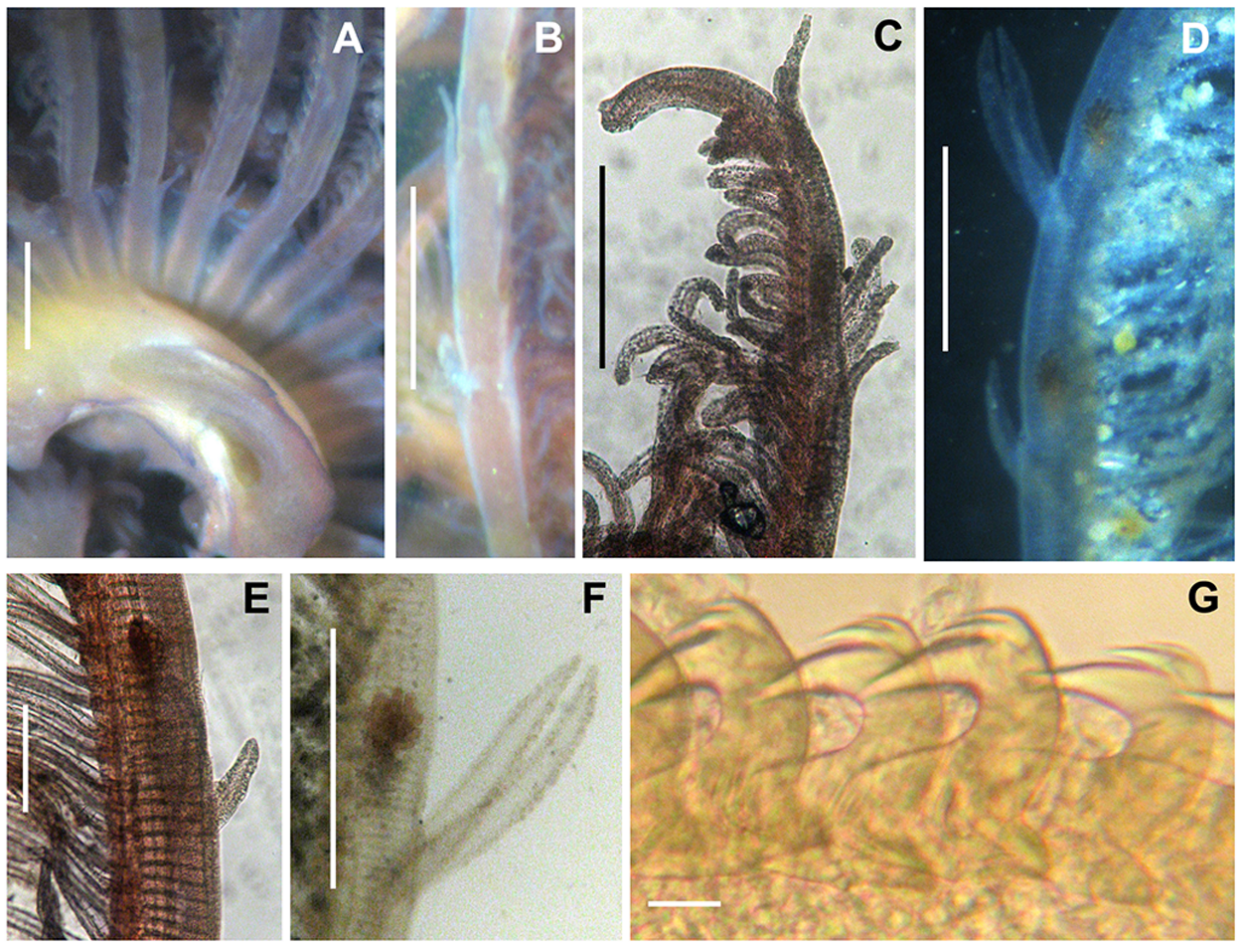
Morphological features of *B*. *boholense* specimens from Indonesia. (A) Branchial lobe showing the unpaired basal stylodes. (B) Detail of microstylodes and macrostylodes along the radiole. (C) Detail of the radiolar tips. (D) Detail of radiole with stylodes (micro and macro) and radiolar eyes. (E) Detail of microstylodes along the radiole. (F) Detail of macrostylodes along the radiole. (G) Detail of thoracic avicular uncini. Scale bars: A-D, F, 500 μm; E, 150 μm; G, 10 μm.

In Fig 10, the morphological features of *B*. *boholense* specimens from its original area of distribution (Indonesia) are shown. Microstylodes appeared digitiform and long as rachis width or slightly longer, while macrostylodes were 2–3 times longer than microstylodes and tongue-like (flattened) in shape (Fig 10B, 10D, 10E and 10F). The basal microstylodes appeared small (as long as rachis width or slightly longer) and unpaired (Fig 10A). Radioles showed short radiolar tips (as long as equivalent space of 6–8 pinnules) (10C) and radiolar eyes appeared small (covering less than 1/2 of side of radiolar rachis at mid-radiolar length) (Fig 10D, 10E and 10F). Thoracic and abdominal uncini showed two rows of teeth over the main fang (Fig 10G).

## Discussion

### Taxonomic status of the Mediterranean taxon

This study revealed significant genetic differentiation in the mitochondrial gene COI between Mediterranean and extra-Mediterranean *Branchiomma* populations, strongly suggesting that they represent two different species. Observed values of pairwise Φ_ST_ and average K2P genetic distance denoted that the two groups of populations belong to two different congeneric species, indeed the levels of genetic distance (K2P) observed between the two groups of populations were similar to those reported for other congeneric polychaete species [18,79–85]. The results obtained from the phylogenetic analysis emphasized the strong separation between the two forms as well: sequences obtained from extra-Mediterranean samples and Mediterranean ones clustered in two distinct and well supported clades.

These results led to a careful re-examination of the morphology of the two groups of samples that were molecularly divergent. The morphological features observed for extra-Mediterranean specimens correspond to those reported for the species *B*. *bairdi* [86]. By contrast, the morphology of the Mediterranean taxon led to presumably reconsider the species *B*. *boholense* for our Mediterranean material originally identified as *B*. *bairdi*. Indeed, Mediterranean specimens showed morphological features comparable to those reported for specimens from Indonesia, reviewed in this paper (Fig 10) and identified as *B*. *boholense* [22] as well as to the syntype examined by Cepeda & Rodríguez-Flores [41]. Besides, the taxonomic characters observed for our Mediterranean material did not coincide with those considered diagnostic for the identification of any other *Branchiomma* taxa, including species native of the Mediterranean area. However, lacking molecular data for the material from Indonesia, it was not possible to corroborate our morphological results with the molecular data during this phase. This approach is highly suggested in the future to validate our findings.

It is worth mentioning that biological data on reproduction and population dynamics concerning the Mediterranean form previously referred as *B*. *bairdi* [29,30] could probably be attributed to *B*. *boholense*. Significant biological differences found between the Mediterranean populations and Mazatlán (Gulf of California) population were previously explained as adaptation to a new environment [29,30]. Results from the present study led to reconsider these biological differences due to the fact that the two examined taxa are actually different species. Indeed, the two species differ in the reproductive features, with a different period and mode of reproduction. Brooding was reported for Malta and Mazatlán populations [26,45], while external fertilization occurs in Mediterranean specimens [30]. Moreover, no sign of asexual reproduction was found in the Mediterranean taxon contrary to what has been reported for *B*. *bairdi* [45]. During the morphological analysis, the presence of the eggs among radioles in the crown of *B*. *bairdi* were often detected, and never observed in the Mediterranean taxon presumably identified in this study as *B*. *boholense*.

#### Morphological distinctiveness

As already reported by different authors [17,22,25,26,37,41,42], *Branchiomma bairdi* and *B*. *boholense* are difficult to distinguish and it is likely that in the past the two species have been frequently misidentified. According to Tovar-Hernández et al. [43], the main distinctive characters separating these two species are the thoracic uncini dentition and the different morphology of macrostylodes: strap-like macrostylodes and 2–3 rows of teeth on thoracic uncini are present in *B*. *bairdi*, whereas *B*. *boholense* has flattened tongue-like macrostylodes and thoracic uncini with one large tooth. However, recently, Keppel et al. [17] and Cepeda & Rodríguez-Flores [41] showed that also in *B*. *boholense* two rows of teeth can be present, suggesting that this character cannot be considered for the separation of the two taxa.

Our morphological observations on Mediterranean and extra-Mediterranean specimens as well as on individuals from Indonesia integrated the results reported by Cepeda & Rodríguez-Flores [41] that compared some Mediterranean material identified as *B*. *bairdi* with the syntype of *B*. *boholense* giving some useful suggestion about the main differences separating the two taxa.

Results from this study suggested that the main discrete morphological differences between the two taxa are the shape of macrostylodes, the development of microstylodes and radiolar eyes, coupled with differences in body size and in the number of teeth over the main fang of thoracic and abdominal uncini. Specimens from Mediterranean populations, presumably to be considered as *B*. *boholense*, showed mainly flattened, tongue-like macrostylodes, short microstylodes (as long as rachis width or slightly longer) and small brownish radiolar eyes (covering less than 1/2 of side of radiolar rachis). By contrast, specimens of *B*. *bairdi* had cylindrical, strap-like macrostylodes, long microstylodes (notoriously longer than rachis width) and big red-pigmented radiolar eyes (covering more than 3/4 of side of radiolar rachis). Moreover, both species showed more than one row of teeth over the main fang. Nevertheless, in *B*. *bairdi* the main fang appeared followed by 2–3 rows of teeth, while in the Mediterranean taxon as well as in specimens from Indonesia (Fig 10G) reviewed in this paper, only two rows of teeth over the main fang seemed to be present.

The slight difference observed between the morphology of the Mediterranean material and the syntype of *B*. *boholense* examined by Cepeda & Rodríguez-Flores [41], could be due to the fact that the syntype was a juvenile (small body size and short, blunt radiolar tips typical of juveniles or regenerating worms), and it was in very bad preserved conditions. In particular, the difference between the two taxa concerning the first, basal stylodes that were reported paired for *B*. *boholense* by Cepeda & Rodríguez-Flores [41], was probably a mistake due to bad preservation, because the distance between the base of the crown and their appearance suggests that they probably referred to the second pair of stylodes that obviously are paired. In addition, well preserved material of *B*. *boholense* from Indonesia also examined in this study (4 adult and 3 juvenile specimens), only present unpaired basal stylodes (Fig 10A). However, Capa et al. [18] already emphasized the presence of basal stylodes being paired in some few taxa (*B*. *lucullanum*, *B*. *bombyx* and *B*. *galei*) and showing plasticity in the majority of the other studied *Branchiomma* species. But the relation of presence-absence of this feature according to juvenile or adult stages, or regenerating worms have not been determined.

As this study shows, two invasive species of *Branchiomma* in the Mediterranean present different reproductive modes and discrete morphological differences, including body size. Capa et al. [18] already highlighted that the traditional diagnostic morphological features of species in the genus *Branchiomma* are greatly homoplastic and polymorphic, and Keppel et al. [17] emphasized that other characters including body size, radiolar crown, dorsal lips and thorax length are not informative, as they are impacted by anaesthetics and fixation methods, or by the regeneration process. Moreover, during the process of morphological identification other difficulties can arise as it is not always possible to work using live specimens or well fixed or preserved samples, and sometimes may not be possible to corroborate identifications using type materials. Six species of *Branchiomma* have been reported as alien worldwide, but it appears that many additional NIS remain undetected for this family, in multiple geographic regions around the world. Consequently, for early detection programs of invasive species we recommend the combined use of molecular and morphological approaches for *Branchiomma* species. It would work at least for *B*. *bairdi*, *B*. *boholense*, *B*. *bombyx*, *B*. *luctuosum* and other seven unnamed, but alien species included in Capa et al. [18]. However, a major revision of type materials is still needed in order to provide the available names for those alien species.

#### Species distribution within the Mediterranean basin

On the basis of both molecular and morphological analysis the present knowledge led to reconsider the distribution of *Branchiomma bairdi* and *B*. *boholense* within the Mediterranean basin.

Both species are present in the Mediterranean area, although, following the results of this study, the previous records of *B*. *bairdi* reported for the Italian coast should probably represent *B*. *boholense* [25,26,27,29,30,87]. Even though *B*. *boholense* seems to have been present in the Eastern basin since the early 1900s [22], recently the species has shown to spread and establish successfully in the central and western Mediterranean basin as shown by its recent reports along the Italian coast [25–27] and in the south-eastern coast of Spain [37]. By contrast, *B*. *bairdi* was actually collected in the islands of Malta and Formentera [26,41], and along the Turkish and Tunisian coasts [38, 40].

The presence of *B*. *bairdi* in the western side of the basin and its records at Canary and Madeira Islands [26,42] is consistent with the probable introduction of *B*. *bairdi* from the Atlantic Ocean throughout the Gibraltar Strait. By contrast, considering the Indo-Pacific origin of *B*. *boholense* and that the first findings of the species are from the eastern side of the basin [22,34], the species might have entered the Mediterranean trough the Suez Canal.

### Genetic structure

The mitochondrial genetic diversity observed within populations of the examined species are comparable to those reported for other polychaetes [88,89]. However, different genetic scenarios are displayed for the two introduced *Branchiomma* species, with the Mediterranean taxon (presumably *B*. *boholense*) showing high levels of genetic diversity, and, otherwise, the introduced Pacific and Atlantic *B*. *bairdi* populations exhibiting low levels of genetic diversity.

Different ranges of genetic diversity have been documented for invasive species according to their life history traits and to the different type of introductions [57,90–93]. As proposed by Voisin et al. [94], invasive species typically present two distinct patterns of genetic diversity: a reduced genetic variability associated to stochastic introductions of a small number of individuals that are more likely subjected to successive bottleneck, or increased levels of genetic diversity due to multiple introductions.

The pattern of genetic diversity of (presumably) *B*. *boholense* observed in the Mediterranean basin is in accordance with the second hypothesis. Indeed, the expansion of this species across the Mediterranean is very recent with possible recurrent introduction events recorded through different years and in localities that are wide apart across the basin [25,26,34,37]. A similar pattern of high genetic variability associated to continuous introductions from different sources was observed in some invasive ascidian species such as *Styela clava* Herdman, 1881 [57] and *Styela plicata* (Lesueur, 1823) along the Italian coasts [92].

This pattern of increased within-population genetic diversity may be also associated to the life history traits of *B*. *boholense*, which showed a short life span, associated with a fast growth, early maturity and a rapid generation turnover ([29] as *B*. *bairdi*). Moreover, this species displays a great ecological plasticity and adaptability in colonizing different habitats and substrates. In particular, among the examined sites around Ischia Island the species was collected also at the Castello Aragonese vent’s system, a venting area where intense CO_2_ emissions lower the seawater pH up to 6.6 values [95] and where the few polychaete species collected in this site respond to stress either by acclimating via phenotypic plasticity (e.g the sabellid *Amphiglena mediterranea*) or through genetic adaptation (e.g. the nereidid *Platynereis spp*) [85,96,97]. *Branchiomma boholense* seems to acclimate to the CO_2_ vent conditions like *A*. *mediterranea*, as the vent-inhabiting populations are not genetically distinct from nearby non-acidified populations. The ecological plasticity of the species coupled with the high genetic diversity observed might in turn enhance its potential of invasion. A connection between high intrinsic genetic diversity and high ecological plasticity was observed also for introduced populations of the Mediterranean species *Sabella spallanzanii* (Gmelin, 1791) in Australian waters [98], where it is considered a pest species [66].

A different scenario of within-population genetic diversity was observed for the studied native and introduced populations of *B*. *bairdi*. As expected, populations sampled from the original distribution range of the species (Tampa Bay and Veracruz) showed very high values of diversity mainly linked to their higher size and stability compared to the introduced ones. Indeed, these populations belong to a taxon with a huge Caribbean distribution, described for Bermuda in 1885, but successively found in Florida [99] and in Veracruz [100–102].

By contrast, the low values of diversity observed in the studied introduced *B*. *bairdi* populations (Madeira, Mazatlán, Galapagos, Hawaii) and their monomorphic condition denoted the occurrence of recent bottlenecks from a single introduction event that accounted for the total loss of the native genetic variability observed. This is also consistent with the recent age of introduction of *B*. *bairdi* in the southern Gulf of California [43] as well as in Galapagos, Hawaii [20,68] and Madeira Island [42]. The recent introduction of this species within Madeira fauna can be inferred considering that Madeira is a site whose fauna is well-known and highly investigated over time [103].

Moreover, Madeira, Galapagos and Hawaii are island populations and, as already observed in other studies [104,105], such populations show lower levels of genetic variation than their mainland counterparts as stochastic introduction events counteract the arrival of new alleles in the gene pool. However, the pattern of diversity observed in the introduced populations of *B*. *bairdi* can be due to the presence of asexual reproduction [45] as well, with the propagation of clones that could have genetically homogenized the population [106,107]. Nevertheless, this low genetic diversity does not seem to affect the successful invasion of the species as already observed for other invasive taxa [57,108].

Pair-wise F_*ST*_ and K2P analysis evidenced a genetic similarity among the Mediterranean populations as well as among the extra-Mediterranean ones, suggesting the presence of a certain relatedness among the Mediterranean populations and between native and introduced *B*. *bairdi* populations as well.

The presence of connections between the different populations of the same species was demonstrated also in the median joining network of haplotypes. The network showed two distinct haplogroups representing Mediterranean and extra-Mediterranean detected mitochondrial haplotypes. In both haplogroups the occurrence of just one haplotype shared by the majority of the individuals confirmed the degree of connections between the Mediterranean populations of presumably *B*. *boholense* as well as between the native and introduced populations of *B*. *bairdi*. As regards the Mediterranean basin, the occurrence of two haplotypes shared by different populations suggested the presence of a low but existing gene flow between these populations likely mediated by local vessels rather than by natural dispersion of the species as supported by the *in vitro* short pelagic larval phase of the species [30]. The Mediterranean basin is highly urbanized and a dense network of harbors and marinas are present along its coasts [109,110], likely acting as sites of secondary dispersal of non-indigenous species [93,111]. Likewise, the relatedness among native populations of *B*. *bairdi* and individuals introduced in the Pacific Ocean and in Madeira Island was demonstrated by the fact that these populations share the same haplotype detected in native populations. This genetic structure corroborates the hypothesis that the introduction of *B*. *bairdi* both in the East Pacific and in the East Atlantic occurred from the Caribbean as already suggested by Tovar-Hernández et al. [45,46] and Ramalhosa et al. [42] for the port of Mazatlán and Madeira Island, respectively. Moreover, this scenario brings attention to the Panama Canal. The Panama Canal is an important point of passage for the maritime traffic with thousands of ships transit the Canal annually [112] and, as already reported by several studies [113–117] it is a potential corridor for the movement of marine species between the Pacific and Atlantic Ocean. It is worth mentioning that haplotype 9 is clearly distinctive from the remaining *B*. *bairdi* samples (average genetic distance, K2P = 0.015) (Figs 2 and 3). No morphological differences were observed and we therefore include this haplotype under *B*. *bairdi*. It comprises samples from Veracruz and Tampa Bay (native range) but also a single individual from the Galapagos Islands (introduced range). We have no information about the underlying causes for the genetic split, but the wide geographic distribution of this haplotype underlines its invasive potential.

In this study, the combined use of both molecular and morphological data allowed to clarify the taxonomic status of the two alien *Branchiomma* species and the genetic structure of the studied populations, providing reliable information on the distribution range of *B*. *bairdi* outside its native range and on the invasion scenario of (presumably) *B*. *boholense* in the Mediterranean Sea.

Further studies with the use of other molecular markers and a bigger pool of samples could corroborate our findings and provide a better understanding of two species invasion process for a complete and exhaustive management program.

## Supporting information

S1 Table Distribution and frequency of COI haplotypes (rows) across the studied populations (columns). (CSV)

S2 Table Pairwise ΦST values of molecular differentiation between samples. Values that were significant are in bold.
(CSV)

S3 Table Average Kimura two-parameter (K2P) distance between the different sampled localities. Values that are significant are in bold.
(CSV)

S4 Table List of specimens utilized for the study with the related GenBank accession numbers and sampling localities. (CSV)
